# High-frequency spinal cord stimulation in failed back surgery syndrome patients with predominant low back pain—single-center experience

**DOI:** 10.1007/s10143-020-01462-5

**Published:** 2021-01-17

**Authors:** Stefan Motov, Kaywan Aftahy, Ann-Kathrin Jörger, Arthur Wagner, Bernhard Meyer, Ehab Shiban

**Affiliations:** 1 Department of Neurosurgery, Technical University Munich, Ismaninger Str. 22, 81675 Munich, Germany; 2 Department of Neurosurgery, University hospital of Augsburg, Augsburg, Germany

**Keywords:** FBSS, Neuromodulation, High-frequency SCS, SCS, Adjacent segment disease, Low back pain

## Abstract

Treatment of patients with failed back surgery syndrome (FBSS) with predominant low back pain (LBP) remains challenging. High-frequency spinal cord stimulation (HF10 SCS) is believed to achieve significant pain reduction. We aimed to evaluate the real-life efficacy of HF-10 SCS in a tertiary spine center. A prospective observational study of all patients with FBSS and predominant LBP who underwent HF-10 SCS surgery was performed between 2016 and 2018. Patients > 18 years with Visual Analogue Scale (VAS) scores of ≥ 5 for LBP and pain duration > 6 months under stable medication were implanted percutaneous under general anesthesia and a trial phase of 7–14 days was accomplished. Primary end point was a successful trial defined as ≥ 50% VAS score reduction for LBP. Thirty-four of 39 (85%) subjects had a successful trial. Fifty-three percent were female and the mean age was 69 years. Median follow-up lasted for 10 months. Devices were removed after a median of 10 months in 5 cases. Remaining 29 patients stated significant VAS score reduction for LBP from 8.1 to 2.9 and VAS for leg pain from 4.9 to 2.2. Twenty-four percent of all patients were able to discontinue their opioids. Eight of 9 patients (89%) with signs of adjacent disc disease and 7 of 10 (70%) patients with hardware failure were successfully implanted with significant VAS reduction for LBP. HF-10 SCS achieves significant pain reduction in most patients with FBSS and predominant LBP. It might be an efficient alternative to revision surgery.

## Introduction

Failed back surgery syndrome (FBSS) is defined as a persistent or recurrent pain, mainly in the lower back and/or legs, even after previous anatomically successful spinal surgery [18]. It is a disabling condition, which affects up to 30% of patients with previous spine surgery [18]. The prevalence of FBSS increases with the number of spinal surgeries performed [17]. FBSS may lead to depression, sleep disturbances, opioid abuse, and dependence as well as burdening socioeconomic problems [16]. Conventional tonic paresthesia-based spinal cord stimulation (SCS) has been applied for decades in patients who suffered of FBSS with predominant neuropathic leg pain with significant positive therapeutic effect. In the last decade, new wave forms like burst stimulation, as well as new modalities like high-frequency (HF) stimulation, were developed for patients with chronic refractory low back pain (LBP) with predominant limb pain [19]. HF-SCS has previously been applied at low amplitudes, in order to remain sub-threshold for sensory activation and paresthesia-free [6]. Currently published studies showed that sub-threshold stimulation at frequencies > 5 kHz can achieve significant pain relief for LBP compared with lower frequencies or sham stimulation [2]. Most of the available data on paresthesia-free HF-SCS originates from randomized multicenter studies with industrial sponsoring [5]. The aim of this study is to provide independent real-world data from a tertiary spine center.

## Methods

A prospective observational study of all consecutive patients with FBSS with predominant LBP (VAS ≥ 5) treated between January 2016 and November 2018 was performed in a single high-volume spine center. The study was performed according to the Declaration of Helsinki and ethical consent was obtained (registration nr. 409/13).

**Fig. 1:**
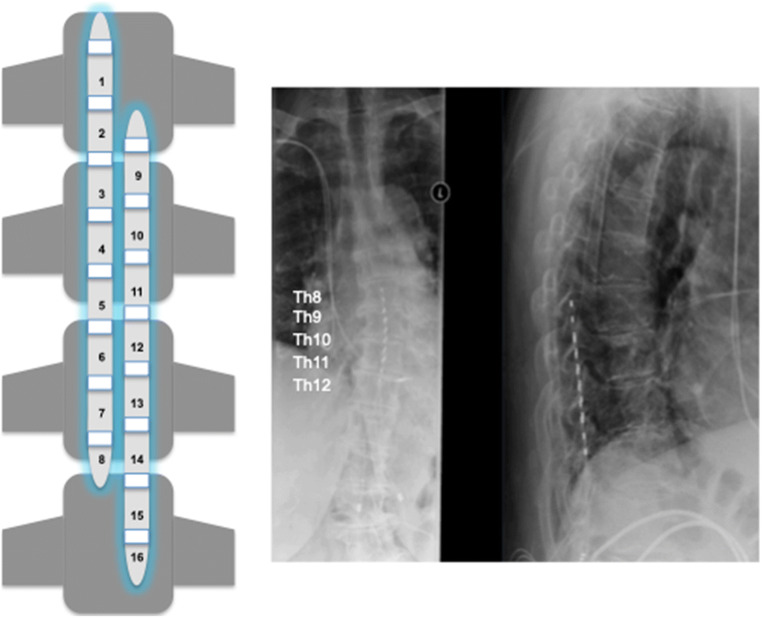
Left: Schematic display of electrode placement. Right: Correct lead placement at Th8–10 level

Inclusion criteria were prior medical history of thoracic or lumbar surgeries with either decompression alone or instrumentation and decompression, at least 6 months of persistent intractable LBP with or without neuropathic component under conservative pain treatment with or without opioids. A psychological assessment was performed preoperatively to rule out major depression, bipolar or psychotic disorder. Exclusion criteria were age < 18 years, no prior spinal surgeries, a causal correlate on spinal imaging (e.g., tumor, infection, or major spinal instability or spinal stenosis related to the patients’ symptoms), and no prior regular pain medication or extensive conservative treatment. Patients meeting all criteria were enrolled in our prospective study and first underwent a trial phase with percutaneous epidural placement of two HF10-SCS leads (10 kHz Senza system, Nevro®) in general anesthesia at the Th8-Th10 level (Fig. 1). Fluoroscopic imaging was performed intraoperatively and on the first postoperative day as well as on follow-up examination. The trial phase lasted for 7–14 days and a successful trial was defined as ≥ 50% pain reduction of VAS scores for LBP. Patients who benefitted from the trial phase were implanted with a permanent implantable pulse generator (IPG, Senza I or II rechargeable IPGs, Nevro®) in general or local anesthesia. The primary end point of this study was a VAS score reduction of ≥ 50% for LBP; secondary end points were discontinuation of opioid medication and rate of surgical complications.

We used SPSS for statistical tests and performed an independent samples *t* test and ANOVA for descriptive analysis and to exploit the differences between the implanted and explanted patients. The chi-square test was further applied for testing significance of parameters.

## Results

### Demographics

**Fig. 2:**
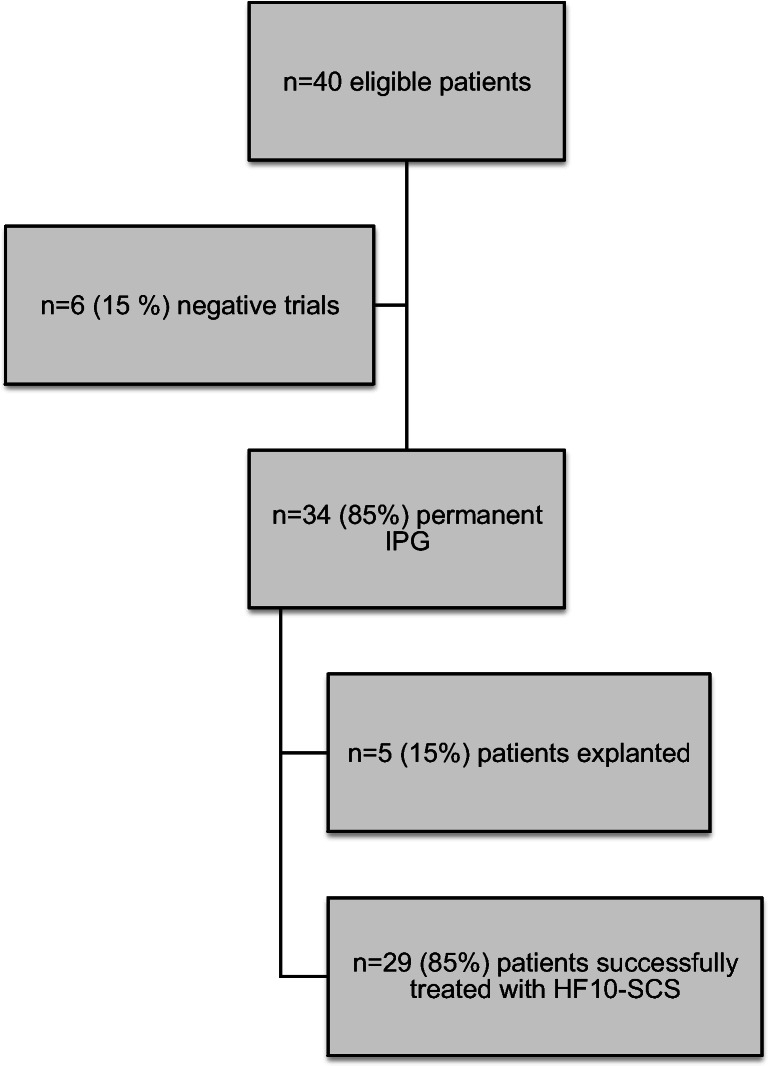
Study patients distribution

In total, 34 of 39 patients (85%) were initially successfully implanted with HF10-SCS after a mean time of 21 days [range 7–84] after initial testing (Fig. 2). Fifty-three percent (*n* = 18) of the implanted patients were female, and 47% (*n* = 16) were male. The mean age was 69 years [range 31–86]. Median age of the implanted patients was 69 years [range 31–86] while the median age of the explanted patients cohort was 72 years [range 49–81] (*p* = 0.669). VAS scores for back and leg pain preoperatively were equally distributed in both groups of implanted and explanted patients (VAS 8.1 back in implanted vs VAS back 7.6 in explanted patients, *p* = 0.418; VAS leg 4.9 in implanted vs VAS leg 7.4 in explanted patients, *p* = 0.054). The percutaneous lead implantation for the successfully implanted patients had a mean duration of 63 min [range 34–107 min] and the IPG surgery a mean duration of 32 min [range 11–94 min]. In patients, who were later explanted due to loss of treatment response, initial leads implantation lasted a mean of 103 min [range 67–138 min], which was statistically significantly longer (*p* = 0.018). IPG implantation in those patients lasted in mean 31 min [range 20–37]. Median follow-up examinations were performed after 10 months [range 4–24] for all patients.

### Trial outcome

In total, 6 patients (15%) experienced a trial failure (Table 1). Although all patients were psychologically assessed before being scheduled for surgery, two patients (5%, patient 1 and 5) suffered from a worsening of their previously described minor depressive disorder after being implanted. One patient (3%, patient 2) lacked even after multiple appointments and phone calls the essential compliance due to a language barrier and could not experience a significant treatment effect. One patient (3%, patient 6) was excluded due to lack of compliance related to progressive Parkinson’s disease with cognitive deficits. The remaining two patients (5%, patients 3 and 4) who experienced a negative trial phase were not able to achieve a sufficient pain reduction even after reprogramming the devices with amplitudes up to 1.5 mA (0.5 mA steps) and applying dual programs with multiple contacts (8 contacts simultaneously) or intervals with on/off stimulation.

**Table 1:** Patients characteristics following a negative trial

Patient	Age (years)	Sex (f=female/m=male)	Previous spine surgery	Trial phase (days)	Comorbidities	Main reason for treatment failure
1	77	f	Multiple surgeries with lumbar stabilization and decompression for DDD L1-L5	17	Depressive disorder under stable medication, alcohol abuse, arterial hypertension, prior hypertensive ICH	Lack of compliance
2	51	m	Discectomy and re-discectomy for disc herniation L4-S1	21	Arterial hypertensive disease, smoking	Insufficient pain reduction
3	89	m	Multiple surgeries with stabilization and decompression for DDD L4-L5	5	Periphery arterial disease, polyneuropathy, smoking, carotid stenosis, renal insufficiency	Lack of compliance
4	68	f	Decompression for spinal stenosis L3-S1	3		Insufficient pain reduction
5	40	f	Stabilization and discectomy for thoracic disc herniation Th11-Th12	4	Depressive disorder under stable medication, smoking	
6	79	m	Multiple surgeries with stabilization L2-L4 and decompression for DDD and spondylolisthesis	15	Parkinson’s disease, arterial hypertensive disease, renal insufficiency	Lack of compliance and insufficient pain reduction

**Fig. 3:**
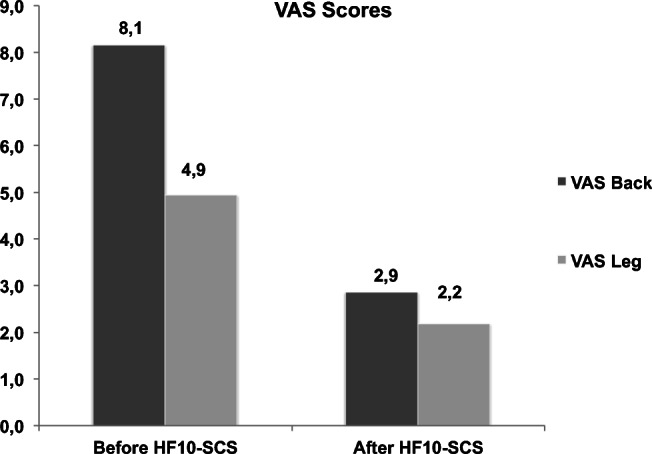
HF10-SCS therapeutic effect based on VAS scores

The implants were removed after a median of 13 months [range 4–36 months] in four cases due to a secondary loss of effect on pain and response to treatment and after 4 months in one case due to a persisting wound healing disorder, which developed over the lead anchors due to a low-grade bacterial infection with streptococcus B specimen. Interestingly two of the explanted patients responded initially excellent to HF10-SCS therapy and lost significant amount of body weight because of being able to resume sports activities. Simultaneously however both of them experienced loss of therapeutic effect after 1 and 3 years respectively even after reprogramming the devices. Lead migration was excluded on X-ray imaging in both cases and analgesics and opioid therapy were adapted to patients’ complaints. However, one of those patients continued to repeatedly complain about pocket pain so revision surgery of IPG was performed and the IPG, which was initially placed in the gluteal area, was transposed in the abdominal fat. As the patient still remained unsatisfied with the surgical result, the device was removed. The other patient was reprogrammed multiple times and later on stated no benefit. There was no significant correlation between prior instrumentation (*p* = 0.574), age (*p* = 0.669), or sex (*p* = 0.798) and the rate of explantation in our patient cohort. The remaining 29 patients (74%) had a significant and persistent pain reduction for LBP from VAS 8.1 to 2.9 and limb pain from VAS 4.9 to 2.2 (Fig. 3).

Further analysis of the study cohort investigating the sagittal imbalance (sagittal vertical axis = SVA > 50 mm) revealed that SVA was pathologically increased in explanted patients and marginal in those with negative trials (median SVA in implanted patients: 37 mm/median SVA in explanted patients: 69 mm/median SVA in patients with negative trial: 47 mm). This might have correlated with a mechanical component of low back pain on axial load in those patients, which potentially exceeded or intensified the neuropathic pain as well. However, it is still debatable whether sagittal imbalance is the cause of low back pain or whether it only correlated with postoperative mechanical complications. Furthermore, 19 of 28 (68%) implanted patients, 4 of 5 (80%) explanted patients, and 4 of 6 negative trials (67%) used to have neuropathic leg pain prior to surgery, which also improved with HF10 SCS therapy (VAS reduction of leg pain from 7 to 3). There were only 4 of 28 (14%) implanted patients and 1 of 6 (17%) with negative trials who were previously treated with discectomy without spinal fusion. Five of 28 (18%) of the implanted patients and 1 of 6 (17%) of those with negative trials suffered from low back pain without neuropathic component, though there was no significant difference for all subgroups considering the lumbar and leg pain outcome with SCS therapy (Table 2).

**Table 2:** Study population characteristics

	VAS back pain preoperatively	VAS back pain with HF10 SCS	VAS leg pain preoperatively	VAS leg pain with HF10 SCS
LBP w/o neuropathic pain	9	3	0	0
Discectomy alone	8,5	2,5	2,5	1,5
Neuropathic leg pain	9	3	7	3
SVA > 50	9	3	0	1
Gabapentin /pregabalin usage	9	3	7	3

### Pain medication

Twenty-five percent (10/40) of the initially recruited patients did not take prior opioid medication before initiation of the HF10-SCS treatment and they neither required any opioids postoperatively. Twenty-four percent (7/29) of the permanently implanted patients were able to completely and permanently discontinue their opioid medication on follow-up examination, relying on NSAID analgesics occasionally. The majority of patients were on gabapentin or pregabalin medication in combination with SCS therapy (13 of 28 successfully implanted patients, 3 of 5 explanted, and 1 of 6 negative trials) due to neuropathic pain. This type of medication was continued by all patients on follow-up.

### Device-related problems

**Fig. 4:**
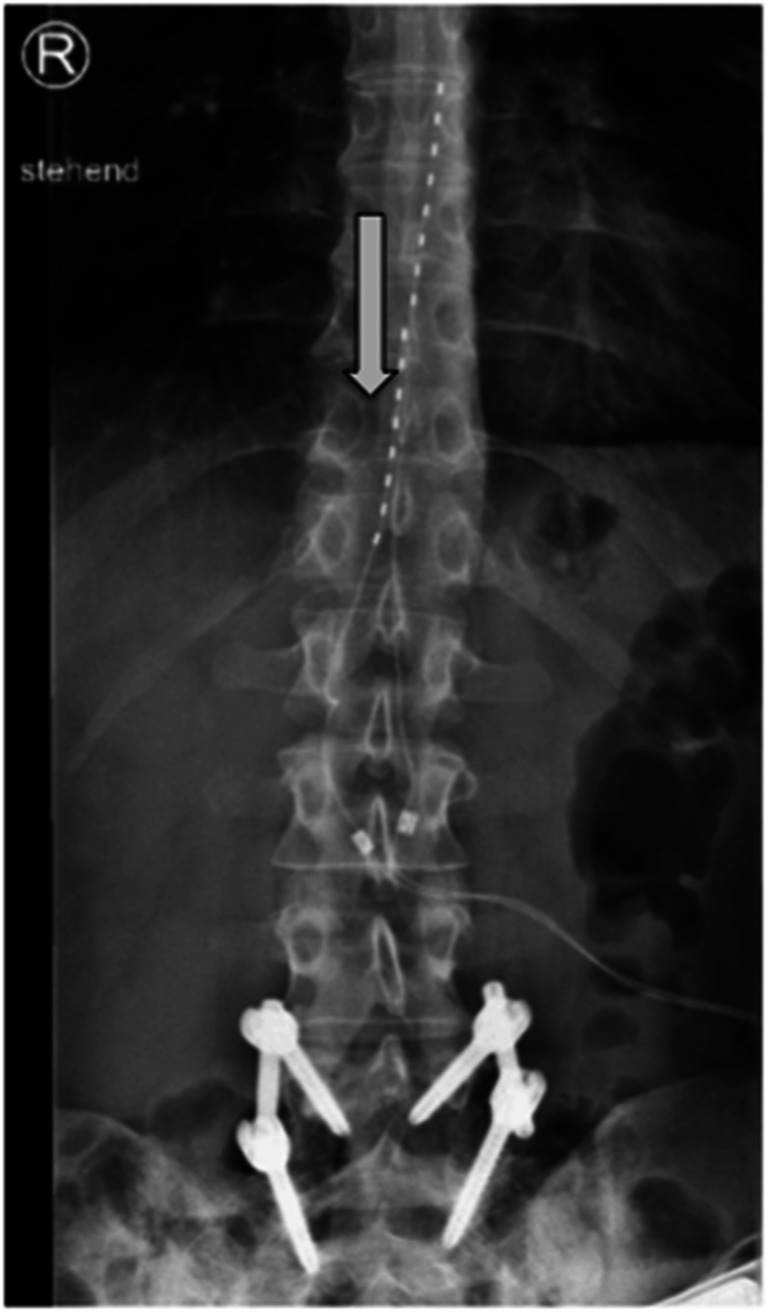
Lead migration on follow-up

**Fig. 5:**
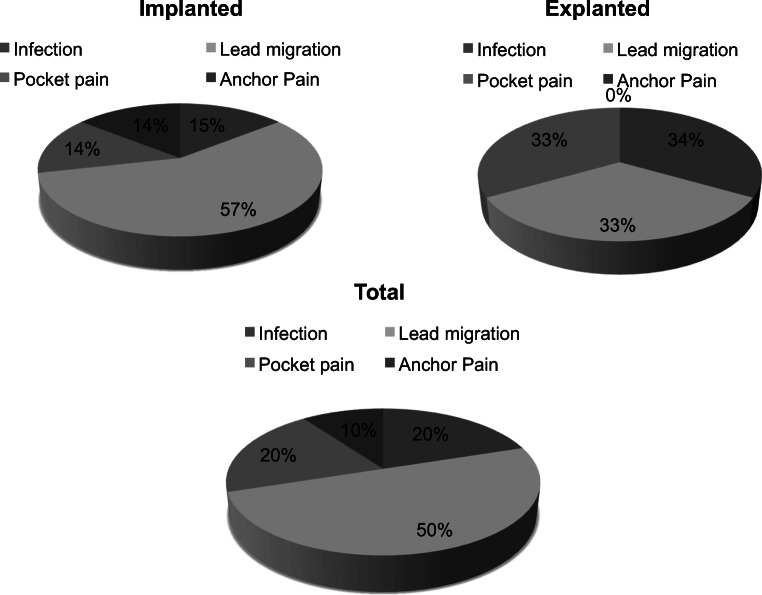
Device-related complications

We monitored patients immediately postoperatively and on follow-up examinations through X-ray imaging in order to detect any hardware failure. Twenty-four percent (7/29) of all permanently implanted patients experienced a lead migration on X-ray imaging during follow-up examinations (Table 3, Fig. 4). There was no significant loss of treatment effect due to lead migration in these cases and no patient required a revision surgery. Two patients (5%) were diagnosed with postoperative infections—one located in the anchoring area and the second one over the IPG. In the first case, we detected a low-grade bacterial infection with Streptococcus B specimen. The second patient was preoperatively already at high-risk with poorly controlled diabetes and complained ten days postoperatively after the IPG implantation of sweating, fever, and purulent discharge of the wound. No bacterial species could be identified in microbiological analysis. Both patients were explanted and treated with intravenous antibiotics for 2 weeks (ampicillin/sulbactam and doxycycline). The latter patient demanded a reimplantation and experienced sufficient pain relieve at follow-up, and the first patient preferred no further surgical treatment. Anchor pain occurred in a single patient (3%) and was managed initially through higher gabapentin dosage and on long-term through a revision surgery in local anesthesia with multiple sutures of the anchors in the muscle fascia, showing a significant symptom relief. Only one of the permanently implanted patients complained about pocket pain and was advised to a revision surgery in local anesthesia with transposition of the IPG above the iliac crest (Fig. 5).

**Table 3:** Device revision characteristics

	Implanted	Explanted	Total
Infection	1	1	2
Lead migration	4	1	5
Pocket pain	1	1	2
Anchor pain	1	0	1

### Adjacent segment disease and hardware failure

**Fig. 6:**
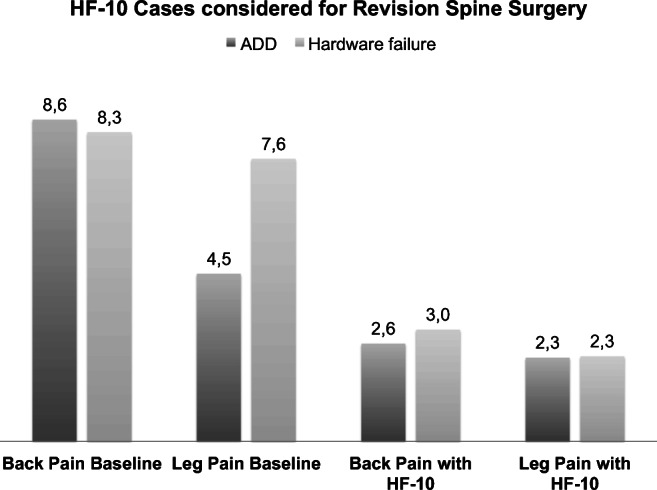
HF-10 cases considered for revision spine surgery

**Fig. 7:**
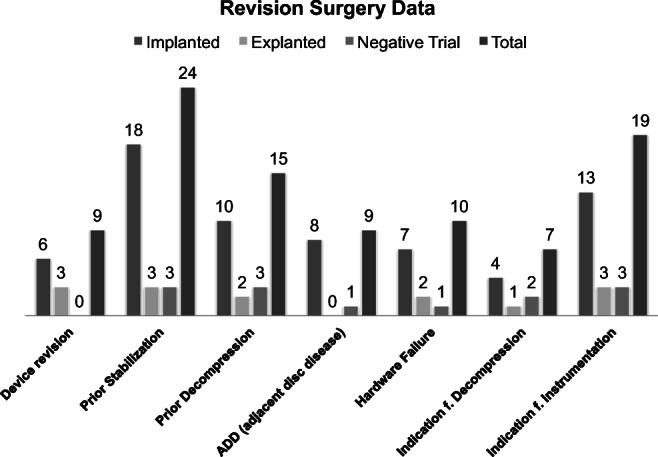
Revision surgery data

**Fig. 8:**
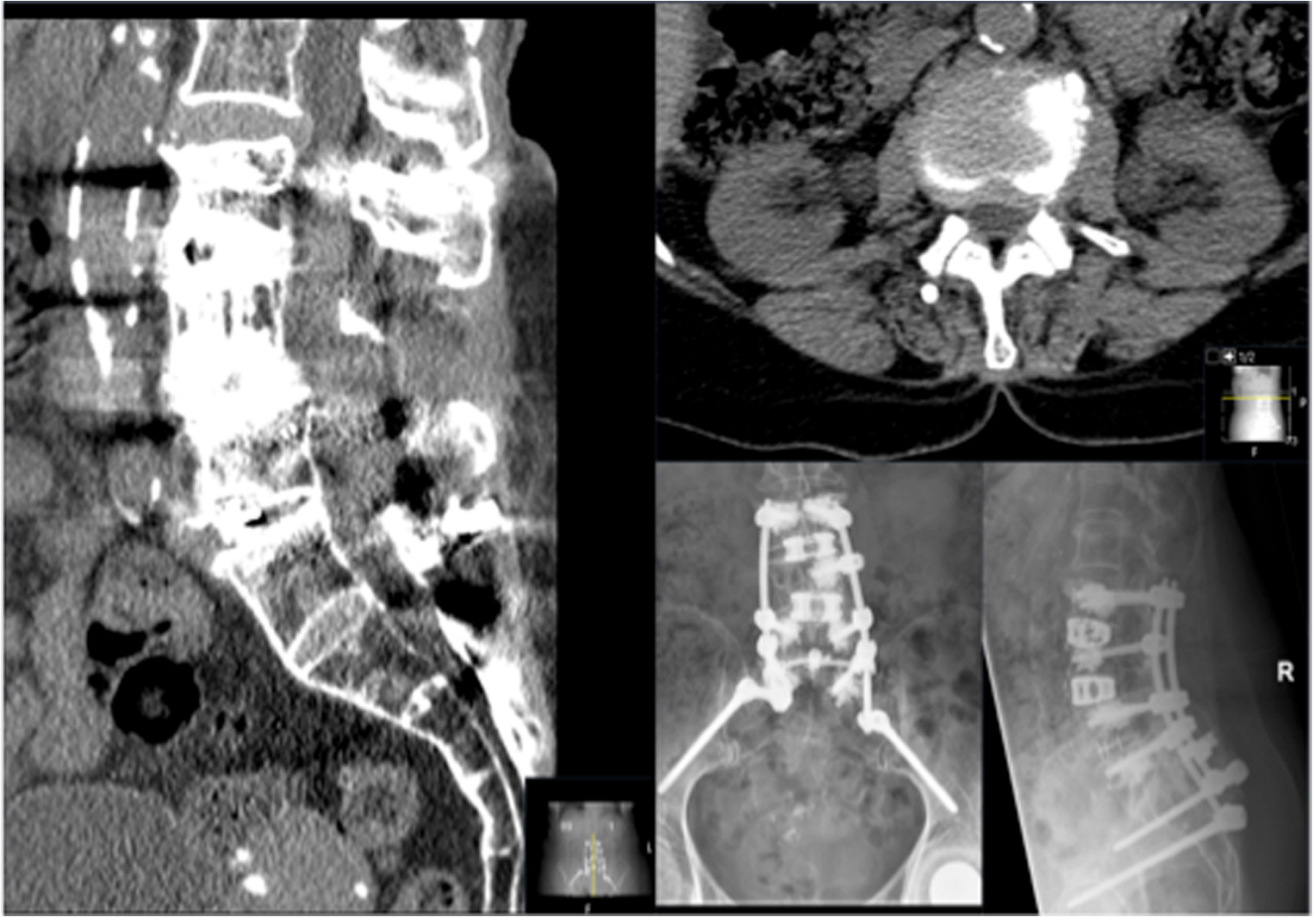
Exemplary case for adjacent disc disease—multiple revision for osteoporotic fractures with adjacent segment disease and no major stenosis

**Fig. 9:**
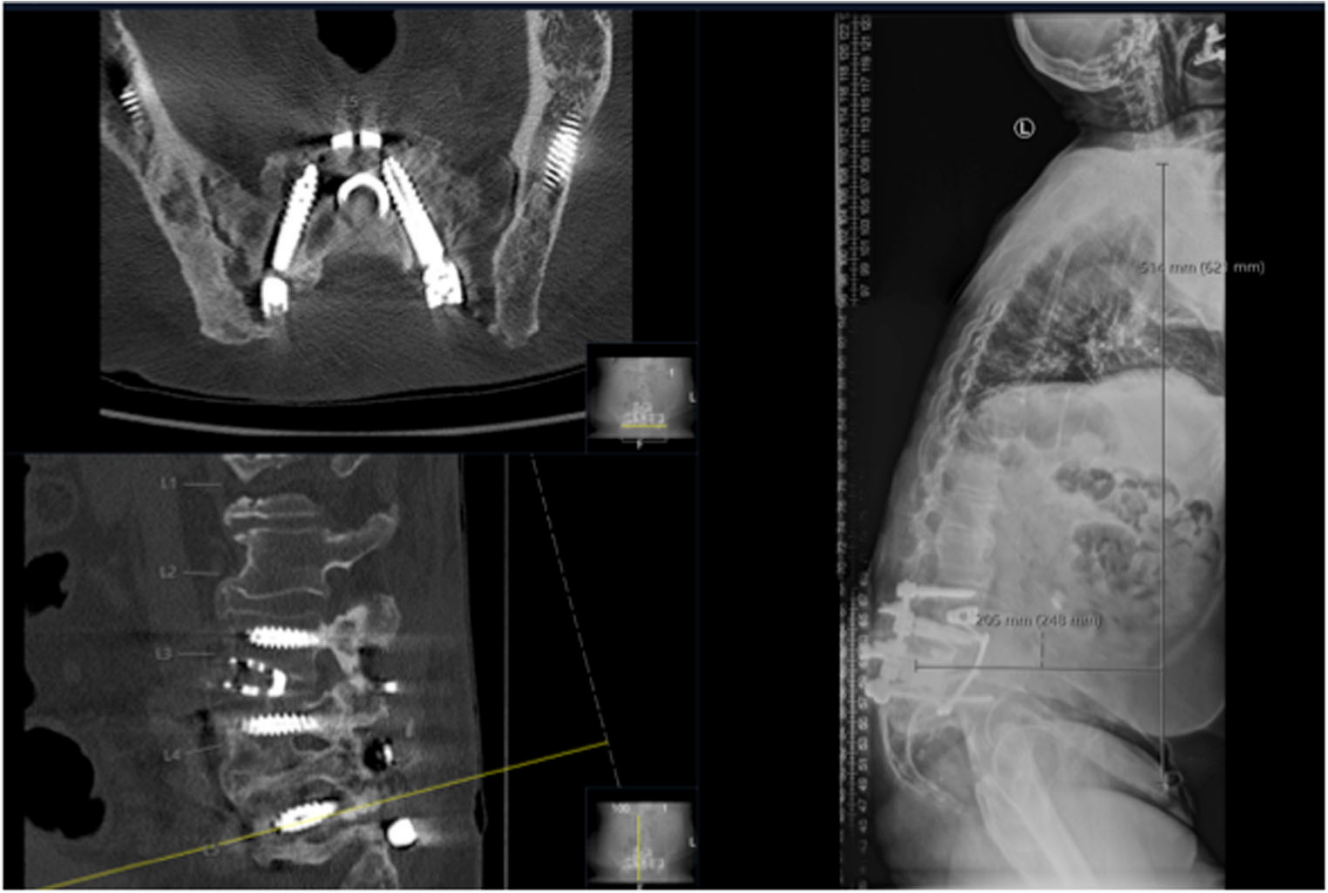
Exemplary case for hardware failure—screw loosening of S1 pedicle screws and pseudarthrosis L5/S1 after multiple revision surgeries for infectious lumbar CSF fistula and initial instrumentation for DDD

Twenty-four patients (62%) had received dorsal or dorsoventral lumbar or thoracolumbar instrumentations and the remaining 15 patients (38%) were treated with discectomies or decompression surgeries prior to SCS therapy (Figs. 6 and 7). Twenty-six patients (6%) of our cohort would normally otherwise have been advised to revision spine surgery—7 patients (18%) for decompression alone and 19 patients (49%) for further extension of the preexisting instrumentation due to adjacent disc disease (ADD) or hardware failure with screw loosening, cage sintering, and/or rod breakage (Table 4, Figs. 8 and 9). In our study, 9 patients (23%) initially presented with persistent LBP (on average VAS score of 9) and radiological signs of ADD in the proximal spinal level without major spinal canal stenosis (Table 5). Eight of 9 (89%) patients were permanently implanted and stated a significant improvement on follow-up examinations with a mean VAS score reduction from 8.6 to 2.6 for LBP and a mean VAS reduction from 4.5 to 2.3 for leg pain (Table 6). Patients diagnosed with hardware failure were identified with heterogenic imaging findings like screw loosening (7 patients, 18%), rod breakage (1 patient, 3%), and non-union with cage sintering (2 patients, 5%). Seven of 10 (70%) patients with hardware failure were successfully implanted and stated a significant improvement of VAS scores for LBP from 8.3 to 3 and VAS score reduction for leg pain from 7.6 to 2.3 (Tables 5 and 6; Figs. 6 and 7).

**Table 4:** Hardware failure data

	Implanted	Explanted	Negative trial
Screw loosening	5	1	1
Rod breakage	0	1	0
Cage sintering	2	0	0

**Table 5:** Revision surgery data

	Device revision	Prior stabilization	Prior decompression	ADD	Hardware failure	Indication f. Decompression	Indication f. Instrumentation
Implanted	6	18	10	8	7	4	13
Explanted	3	3	2	0	2	1	3
Negative trial	0	3	3	1	1	2	3
Total	9	24	15	9	10	7	19
Percent	23%	62%	38%	23%	26%	18%	49%

**Table 6:** HF-10 efficacy in ADD and hardware failure

	ADD	Hardware failure
Back pain baseline	8.6 ± 0.9	8.3 ± 1
Leg pain baseline	4.5 ± 4.8	7.6 ± 1.6
Back pain with HF-10	2.6 ± 1.5	3 ± 1.2
Leg pain with HF-10	2.3 ± 1.5	2.3 ± 1.3

## Discussion

A sustainable and effective pain reduction was achieved in all implanted patients for back and leg pain over a mean follow-up period of 10 months. Constant pain reduction of 64% for LBP in 85% of all patients was observed. This real-world data is similar to the published results from previous randomized controlled trials [3, 10]. To date, neuromodulation is indicated in neuropathic LBP without a clear mechanical pain that may be attributed to the patient’s symptoms [11]. In our patient cohort, neuropathic pain was not considered a mandatory inclusion criterion for test trial enrolment. In contrast to multiple revision surgeries with re-discectomies or extensive instrumentations, which might also have been suitable for some of our patients, we maintained a high back pain improvement with very low complication rates (2.5% infection rate, no postoperative hematoma or broken leads on follow-up, no significant therapy loss despite lead migration) and short surgical times. In a recent meta-analysis of 37 studies with 1483 patients, Dower et al. showed that although greater improvement of back pain was achieved in patients undergoing re-discectomy and fusion compared to re-discectomy alone, the rate of a satisfactory outcome was similar in both groups [8]. Indeed patients who undergo stabilization surgeries are exposed to higher risks for reoperation than patients who receive decompression alone [9]. With increasing numbers of fusion and decompression surgeries in the last two decades, the number of FBSS patients without radiological correlate grows tremendously. Revision surgeries are not necessarily associated with improved pain scores, but have a higher rate of complications including increased bleeding, infections, acute respiratory distress syndrome, longer hospital stays, and higher mortality rates than the primary surgeries [7]. According to the randomized controlled trial (RCT) by North et al. comparing SCS to further surgical interventions (decompression alone or decompression with fusion), the mean costs per successful outcome for revision surgery were on average $57,571 higher than a successful randomization to SCS therapy, which emphasizes the cost-efficiency of SCS implantation [15]. In addition, North et al. also demonstrated in a level I RCT that conventional SCS therapy is more effective than repeated surgery for a subpopulation of FBSS patients [14]. However, North et al. did not apply HF10-SCS therapy on their patient cohort, which is supposed to be even more effective on the long-term for FBSS patients with LBP. Kapural et al. demonstrated in the SENZA trial (level I RCT) a higher effectiveness of HF10-SCS compared to tonic low-frequency SCS providing higher responder rates for leg (83%) and back (85%) pain [10]. A recent review about treatment options for FBSS patients by Amirdelfan et al. indicates that there is currently no level I evidence for success of revision spine surgery in those patients. Moreover, the only two existing level II studies showed no significant differences in pain and disability scores or Oswestry Disability Index (ODI) between a surgical intervention and conservative options [4]. According to our results, HF-10 SCS appears to be a viable option for patients with mechanical low back pain.

Interestingly we experienced significantly longer surgical times (103 min vs 70 min) for percutaneous epidural lead placement in patients who were later explanted. Even though X-ray imaging in those cases confirmed correct lead placement intra- and postoperatively, an extensive scar tissue in the spinal canal due to prior surgeries might have led to lead placement difficulties and later therapy loss.

Treatment of patients with concomitant degenerative spine disease and Parkinson’s disease remains very challenging. In our series, two patients with Parkinson’s disease were ultimately non-responders to HF-10 therapy. One patient had a negative test trial (patient 6) and the second patient experienced loss of initial treatment effect following 3 months after IPG implantation. In recent literature, there are only a few case reports and case series dealing with SCS treatment in patients with Parkinson’s disease. However, Parkinson’s should not be an exclusion criterion as several patients reportedly improved in walking posture, rigidity, and pain intensity through spinal cord stimulation [8, 9].

In our study, a high number of patients (24%) were able to discontinue their opioids on follow-up. Those are similar results to larger studies like the SENZA trial, in which over one-third of all subjects who received HF10-SCS therapy were able to reduce or wean off their opioids despite an average of 13 years of prior chronic pain [13].

We did not encounter any severe mechanical complications, e.g., lead fracture or disconnection, which has a reported incidence between 5 and 9%. We did not observe clinically evident treatment loss due to lead migration even though on follow-up imaging we detected a moderate lead migration in 24% of all permanently implanted patients. Lead migration has a reported incidence in the literature between 0 and 27% [19]. These hardware complications can be minimized by using appropriate leads, anchoring, and suturing techniques. Kapural et al. demonstrated in SENZA-RCT that significant lead migration requiring intervention in both the HF10-SCS and the traditional SCS arms occurred in only less than 5% of all cases [10].

Several studies have demonstrated the benefit of novel SCS therapies over traditional low-frequency SCS for the treatment of patients with chronic low back and/or leg pain. SENZA-RCT showed that paresthesia-free HF10-SCS was superior to low-frequency stimulation for treatment of chronic low back pain with leg pain. The SUNBURST crossover trial recently found that high-frequency burst stimulation was preferred over low-frequency tonic SCS with patients citing better pain relief and a preference for paresthesia-free SCS [12]. However, it is impossible to state whether some patients experienced pain relief due to placebo effect since high-frequency neuromodulation works paresthesia-free. HF10-SCS blocks large-diameter fibers from producing action potentials, which mostly carry information of vibration and pressure, and hence would avoid inducing paresthesia. The same stimulation activates medium- and smaller-diameter dorsal column fibers, which leads to spinal pain inhibition through gate control mechanisms [1]. However, no published study has inferred potential neurophysiological or neurochemical changes that may occur subtly and slowly but progressively after paresthesia-free HF-SCS as for activation of wide dynamic range (WDR) neurons or suppression of Aβ-fibers (supra-threshold) and synaptic transmission of C-fibers [6].

## Conclusion

HF10-SCS therapy is able to achieve highly satisfactory pain reduction in most patients with FBSS with predominant mechanical LBP. In summary, HF10-SCS therapy may contribute to an efficient, cost-effective, and less invasive alternative to revision spine surgery in patients with LBP without compression of neural structures or apparent instability.

## Data Availability

The datasets used and/or analyzed during the current study are available from the corresponding author on reasonable request.
